# Serum iodine concentration and its associations with thyroid function and dietary iodine in pregnant women in the southeast coast of China: a cross-sectional study

**DOI:** 10.3389/fendo.2023.1289572

**Published:** 2023-11-09

**Authors:** Shumi Ji, Xiaoyan Wu, Jiani Wu, Diqun Chen, Zhihui Chen

**Affiliations:** Department of Endemic Diseases, Fujian Center for Disease Control and Prevention, Fuzhou, China

**Keywords:** pregnant women, iodine nutrition, serum iodine, urinary iodine, dietary iodine, thyroid diseases, thyroid function

## Abstract

**Background:**

Iodine deficiency is a major public health problem in pregnant women. Serum iodine (SI) may represent a useful biomarker for iodine nutrition evaluation. We aimed to assess the relationship between serum iodine concentration (SIC) and urinary iodine concentration (UIC), dietary iodine, thyroid function, and thyroid diseases in pregnant women in the southeast coast of China, and to provide a normal reference range of SIC for pregnant women.

**Methods:**

A multistage random sampling method was used to select the study population. We collected urine and blood samples from pregnant women and determined UIC and SIC as well as thyroid function using Arsenic-Cerium Catalytic Spectrophotometry, inductively coupled plasma mass spectrometry, and Beckman Coulter Access2 chemiluminescent immunoanalyzer and kit, respectively, and administered a questionnaire on dietary iodine intake in pregnant women.

**Results:**

There was a significant negative correlation between SI and thyroid-stimulating hormone (TSH) (*r* = −0.141) and a significant positive correlation between SI and free triiodothyronine (FT_3_) (*r* = 0.106), free thyroxine (FT_4_) (*r* = 0.236), triiodothyronine (TT_3_) (*r* = 0.229), total thyroxine (TT_4_) (*r* = 0.433), and dietary iodine intake (*r* = 0.068). There was a significant difference in SI levels of pregnancy between the second (78.13 μg/L) and third trimester (75.37 μg/L) (*p* = 0.018). SI levels between inadequate intake (74.58 μg/L) and appropriate intake (77.92 μg/L) groups were statistically different (*p* = 0.036). Low SIC was a risk factor for the development of hypothyroxinemia (adjusted OR = 3.14, 95% confidence interval: 1.75–5.66). The reference range for SIC in normal pregnant women is 45.03–112.44 μg/L.

**Conclusion:**

SI may be a composite indicator of iodine nutritional status and thyroid function.

## 1 Introduction

Iodine is one of the essential trace elements in the human body and is the main component in the synthesis of thyroid hormones. Deficiency or excess of iodine can be harmful to human health (1). Nearly 2 billion people worldwide still suffer from inadequate iodine intake (2). China used to have a high prevalence of iodine deficiency disorders (IDDs) (3).

Iodine deficiency, especially in pregnant women, is a major public health problem (4, 5). In the 1990s, China adopted universal salt iodization (USI) as the national strategy (6). After years of mandatory USI, China has virtually eliminated IDDs (7). Therefore, public health concern has shifted toward mild iodine deficiency (median urinary iodine concentration [UIC] was 50–99 μg/L in adults) to moderate iodine deficiency (median UIC was 20–49 μg/L in adults), which remains prevalent in many regions (1, 8, 9), especially among pregnant women. Owing to their special physiological status, pregnant women have a 50% higher iodine requirement than non-pregnant adults and are at increased risk of iodine deficiency (10). IDDs in pregnant women lead to increased miscarriage and infant mortality (11). Adequate dietary iodine intake is therefore particularly important for women during pregnancy, and the need for iodine nutritional assessment of pregnant women is even more urgent.

Because of widespread iodine deficiency or some cases of iodine overexposure, iodine biomonitoring is important, but there is no established biomarker for individual iodine status (12). Considering that serum iodine (SI) levels exhibited much less individual variation (13) and it is the biomonitoring approach being closest to the bioavailable I^−^ supply for the thyroid gland (14), SI may, therefore, represent a better and important biomarker for iodine nutrition evaluation (13). SI has been reported to be valuable in the diagnosis of thyroid diseases (15). Therefore, the timely detection of abnormal SI levels may predict the risk of clinical or subclinical thyroid diseases. However, there are fewer studies on the relationship between SI and dietary iodine, thyroid function, and disease in pregnant women. There are no standards for reference ranges for normal human SI in China; only some reference ranges have been proposed by renowned foreign laboratories Mayo Clinic, Quest Diagnostics, and World Health Organization (WHO), and they do not differentiate between different groups of people, so there is still a lack of uniform standards for normal reference ranges in China (16).

Fujian Province is on the southeast coast of China, and previous studies have shown that coastal pregnant women are still mildly deficient in iodine nutrition (17). This study aims to evaluate the relationship between SI and urinary iodine (UI), dietary iodine intake, thyroid function, and thyroid diseases in pregnant women, and to provide a normal reference range for SI in pregnant women in the southeast coastal region. The study will provide more evidence for the evaluation of iodine nutrition status and iodine metabolism processes in pregnant women, and provide further scientific reference for the current USI policy in China.

## 2 Subjects and methods

### 2.1 Study population

**Figure 1:**
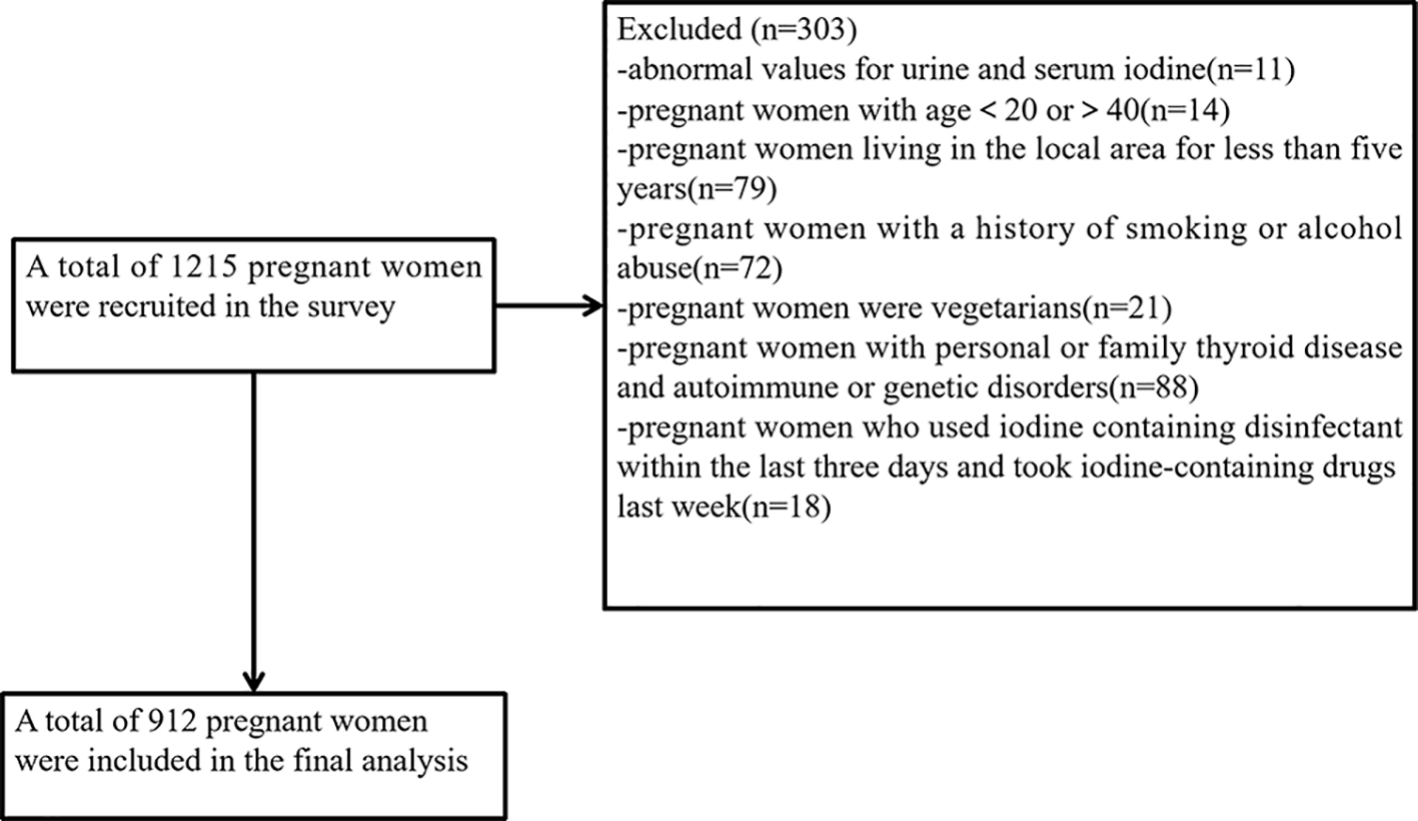
Flowchart of the participants in the study.

This cross-sectional study employed the multistage cluster sampling method in 2020. Data collection was conducted from 1 June 2020 to 30 September 2020. The selection of survey sites was described in detail in our previous study (17). One urban and one rural survey site were selected from each of the nine administrative divisions in Fujian Province, making a total of 18 survey sites. In each monitoring county, five towns were randomly selected from five different directions (east, west, south, north, and west). A certain number of pregnant women were selected from each township (insufficient numbers could be filled in neighboring towns). All subjects were asked to complete questionnaires that included demographic information (e.g., name, age, gestational week, height, weight, etc.), medical history (e.g., endocrine system diseases), smoking, alcohol abuse, and family history of thyroid and other endocrine disorders. The participants from the villages were recruited, and the inclusion criteria were as follows: (1) previously healthy, no history of thyroid diseases, no history of autoimmune disease, no history of endocrine disease, no family genetic disorders, etc.; (2) age between 20 and 40 years old and have lived in the local area for 5 years or more; and (3) no special dietary habits, e.g., vegetarian diet. The exclusion criteria were as follows: (1) those with a history of smoking or alcohol abuse; (2) those who have used iodine-containing lotions in the last 3 days, those who have taken cydiodine tablets, amiodarone hydrochloride, or those who have had recent imaging tests. A total of 1,215 pregnant women were surveyed in this study. Based on the inclusion and exclusion criteria and the exclusion of 9 abnormal values for urine samples and 2 abnormal values for blood samples, a total of 912 pregnant women were eventually included in the statistical analysis. The flowchart is presented in **Figure 1**.

### 2.2 Sample collection

No less than 5 mL of fasting spot-urine sample, 5 mL of fasting venous blood, and 50 g of edible salt were collected from each participant in the morning (between 8:00 and 11:00) to test iodine levels and thyroid function without determining creatinine. Urine samples from all subjects were kept in clean plastic tubes at 4°C. Blood samples are stored at a low temperature (−20°C). Salt samples should be sealed in plastic bags and protected from sunlight. Urine and salt samples should not be mixed. Water samples were collected on a township basis and stored in iodine-free treated polyethylene plastic bottles at 4°C. All samples collected will be sent to the Centre for Disease Control and Prevention for testing. Dietary surveys and biological sample collection were carried out simultaneously. Food Frequency Questionnaire (FFQ) was used to investigate the dietary iodine intake of pregnant women. We evaluated the iodine concentration in food with reference to the China Food Composition Tables (Standard Edition 6th edition) (18). We selected foods whose iodine content is more than 10 μg/100 g, and dietary iodine contribution is more than 1% (19). Food items surveyed included staple food groups, soya bean products, meat, eggs, dairy products, aquatic products, fruits and vegetables, offal, snacks, nuts, beverages, and condiments. The food frequency (times) was divided into ≥1 time/day, 1–6 times/week, 1–3 times/month, 1–5 times/half-year, and not eat (19). With reference to the color charts of foods in China Food Composition Tables (18), the professional investigators asked the participants about the frequency and food consumption. Drinking water intake was recorded. Dietary iodine intake (μg/day) = Σ (intake of each type of food × iodine content of each type of food) + amount of drinking water × iodine content of water + amount of iodinated vitamins × iodine content of vitamins + salt intake × salt iodine content × (1–20%), 20% being the cooking loss rate of iodized salt as defined by WHO (20).

### 2.3 Determination methods

The samples were processed in the laboratory of Fujian Provincial Center for Disease Control and Prevention and their UIC was determined by Arsenic-Cerium Catalytic Spectrophotometry (21), salt samples were tested by the general test method of the salt industry (22), water iodine was detected by the method recommended by the National Reference Laboratory of Iodine Deficiency Disorders (23), and serum iodine concentration (SIC) was detected by inductively coupled plasma mass spectrometry (24). A Beckman Coulter Access2 chemiluminescent immunoassay analyzer and kit were used to measure eight indicators of serum thyroid function: thyroid-stimulating hormone (TSH), thyroglobulin (Tg), free triiodothyronine (FT_3_), free thyroxine (FT_4_), total triiodothyronine (TT_3_), total thyroxine (TT_4_), thyroid peroxidase antibody (TPOAb), and thyroglobulin antibody (TGAb). Specially trained technicians performed thyroid ultrasonography. We used GE LOGIQ BOOK XP manufactured by General Electric Medical Systems (China) with a 7.5-MHz probe to measure the thyroid volume, nodule diameter. The thyroid volume was calculated according to 0.479 × (left lobe: length ×width × thickness + right lobe: length × width × thickness) (length, width, and thickness in the equation used the unit of cm, and thyroid volume used the unit of mL).

### 2.4 Chemicals and instrumentation

Principles, instruments, and chemicals for iodine measurement are described detailly in the **Supplementary File** entitled “Chemicals and instrumentation for iodine measurement”. Iodine measurements of all samples were performed at Fujian Province Center for Disease Control and Prevention and met the quality control requirements of the National Reference Laboratory for Iodine Deficiency Disorders. We use nationally certified reference Standard Substances for quality control. Only when all measured values of the reference substance are controlled can the test results be accepted. The standard curve’s correlation coefficient had to be greater than 0.999 (25).

### 2.5 Diagnostic criteria

The gestational week was determined based on the time of the last menstruation (<13 weeks was defined as the first trimester (T1), 13–27 weeks was the second trimester (T2), and ≥28 weeks was the third trimester (T3) (26). UI in pregnant women <150 µg/L is considered insufficient, 150–249 µg/L is considered adequate, 250–499 µg/L is considered above requirements, and ≥500 µg/L is considered excessive (1). According to the Reference Intake of Dietary Nutrients for China Residents (27), iodine intake less than 160 μg/day is defined as insufficient iodine intake, 160–600 μg/day is defined as appropriate iodine intake, and more than 600 μg/day is defined as excessive iodine intake. The recommended iodine intake for pregnant women is 230 μg/day. TSH and FT_4_ were categorized according to gestation period (T1–T3). The normal reference ranges (with 95% confidence interval [CI]) for the test kits (Beckman) used for TSH (in mIU/L) and FT_4_ (in pmol/L) according to trimester (T1, T2, and T3) were as follows: 0.03–4.00, 0.35–3.86, and 0.46–4.82 mIU/L for TSH and 9.54–16.09, 7.33–12.07, and 6.40–11.21 pmol/L for FT_4_, respectively. Thyroid diseases were diagnosed according to the Guidelines for the Diagnosis and Management of Thyroid Disease during Pregnancy and the Postpartum (28). Diagnostic criteria for thyroid disorders in pregnant women are summarized in **Supplementary Table 1**. TPOAb was considered positive with values >9 IU/mL and TGAb with values >4 IU/mL by the test kits (Beckman). Thyroid ultrasonography was performed according to Chinese health standards and the normal female thyroid volume is ≤18 mL (29) and thyroid nodule is one or more nodule (>5 mm) without goiter (30).

### 2.6 Statistical analysis

WPS (Beijing and Zhuhai Kingsoft Software Company) and SPSS version 24 (IBM, Armonk, NY, USA) were used to collate and analyze the data. The Kolmogorov–Smirnov test was used to test whether the data conformed to a normal distribution. Non-normally distributed data were reported as median (M) and interquartile range (IQR, P_25_–P_75_). We calculate P_2.5_ to P_97.5_ as the 95% medical reference range for the non-normal data. The correlation between the non-normally distributed two sets of variables was analyzed using Spearman’s correlation. We used Curve Estimation fitting model to test a trend between the two sets of data and chose the most appropriate model based on the *R* ^2^. The Mann–Whitney *U* test was used for comparison between two non-normal data sets, and the Kruskal–Wallis *H* test was used for comparison between multiple non-normal data sets. Normally distributed data were expressed as mean ± standard deviation (SD), and 95% medical reference range of information was calculated using mean ±1.96×SD. Analyses of variance (ANOVAs) were used for comparisons between multiple groups of normal data, and LSD tests were used for two-way comparisons. Maternal SIC less than the 10th percentile is defined as low SIC and that greater than the 90th percentile is defined as high SIC (31). We used receiver operating characteristic (ROC) curves to determine the diagnostic value of SIC and UIC. The closer the area under the ROC curve is to 1, the higher the diagnostic value. Set *p* < 0.05 as statistically significant.

### 2.7 Ethics approval and consent to participate

This study was conducted according to the guidelines laid down in the Declaration of Helsinki and all procedures involving human subjects/patients were approved by the Medical Ethics Committee of Fujian Provincial Center for Disease Control and Prevention (No. 2020032). Written informed consent was obtained from all subjects/patients.

## 3 Results

### 3.1 Description of the basic characteristics

Basic characteristics of pregnant women at different trimesters are shown in **Table 1**. This included a total of 215 (23.6%) women in the first trimester, 458 (50.2%) in the second trimester, and 239 (26.2%) in the third trimester. After testing for normality, age, height, weight, and BMI were non-normally distributed and the results were expressed as medians and quartiles; the Kruskal–Wallis *H* test was used for comparison between groups. Differences in age and height between trimester groups were not statistically significant. Weight and BMI were highest in the third trimester. There were significant differences in body weight and BMI between trimester groups (*p* < 0.0001).

**Table 1:** Basic characteristics of pregnant women at different trimesters.

Variables	Overall	T1	T2	T3	*p*	*H*
	*n* = 912	*n* = 215	*n* = 458	*n* = 239		
**Age (years)**	30 (27, 30)	30 (26, 33)	30 (27, 32)	30 (26, 33)	0.835	0.361
**Height (m)**	1.58 (1.55, 1.62)	1.58 (1.55, 1.62)	1.59 (1.55, 1.63)	1.58 (1.55, 1.62)	0.587	1.067
**Weight (kg)**	58 (52, 65)	53 (49.9, 60)	57 (51.5, 64)	64 (59, 70)	<0.0001**	131.03
**BMI (kg/m^2^)**	22.89 (20.83, 25.68)	21.22 (19.56, 23.56)	22.60 (20.66, 25.23)	25.24 (23.44, 27.51)	<0.0001**	146.07

BMI, Body mass index; T1, First trimester; T2, Second trimester; T3, Third trimester.

All values are reported as median (M) and interquartile range (IQR, P_25_–P_75_).

Significance between different trimesters using the Kruskal–Wallis H test. H is the test statistics.

**p < 0.0001.

The bold values mean that the p value is less than the set p value, and the difference is statistically significant.

### 3.2 Iodine nutrition index

SIC, UIC, and iodine intake in pregnant women at different trimesters are provided in **Table 2**. After the normality test, UIC, SIC, and iodine intake showed non-normal distribution, the results were expressed by median and quartile, and the Kruskal–Wallis *H* test was used for comparison among groups. The difference in UIC between trimester groups was not statistically significant (*H* = 1.299, *p* = 0.522). The median value of SIC in pregnant women was the lowest in the third trimester and the highest in the second trimester. Median UIC of pregnant women in all trimesters was less than 150 μg/L, which can be classified as iodine insufficiency according to the WHO criteria. We found a significant difference in SI levels of pregnancy between the second (78.13 μg/L) and third trimester (75.37 μg/L) (*p* = 0.018). Iodine intake during pregnancy is less than the recommended iodine intake of 230 μg/day. There was no significant difference in iodine intake between trimester groups (*H* = 0.017, *p* = 0.992).

**Table 2:** Serum iodine concentration (SIC), urinary iodine concentration (UIC), and iodine intake in pregnant women at different trimesters.

Variables	Overall	T1	T2	T3	*p*	*H*
	*n* = 912	*n* = 215	*n* = 458	*n* = 239		
**UIC (μg/L)**	148.40 (104.22, 217.65)	148.18 (101.1, 217.63)	149.55 (109.72, 222.01)	147.33 (99.36, 216.10)	0.522	1.299
**SIC (μg/L)**	77.17 (68.86, 87.31)	76.91 (65.67, 87.77)	78.13^‡^ (68.39, 88.21)	75.37 (63.19, 85.43)	0.023*	7.552
**Iodine intake (µg/day)**	220.57 (159.11, 303.32)	224.59 (151.96, 302.15)	216.23 (160.45, 308.25)	222.80 (157.45, 255.19)	0.992	0.017

SIC, Serum iodine concentration; UIC, Urinary iodine concentration; T1, First trimester; T2, Second trimester; T3, Third trimester.

All values are reported as median (M) and interquartile range (IQR, P_25_–P_75_).

Significance between different trimesters using the Kruskal–Wallis H test. H is the test statistics. p-value significance set at p < 0.05.

‡Significant differences compared to T3 (H = 57.636, p = 0.018).

*p < 0.05.

The bold values mean that the p value is less than the set p value, and the difference is statistically significant.

### 3.3 Correlation analysis of factors influencing SI

**Figure 2:**
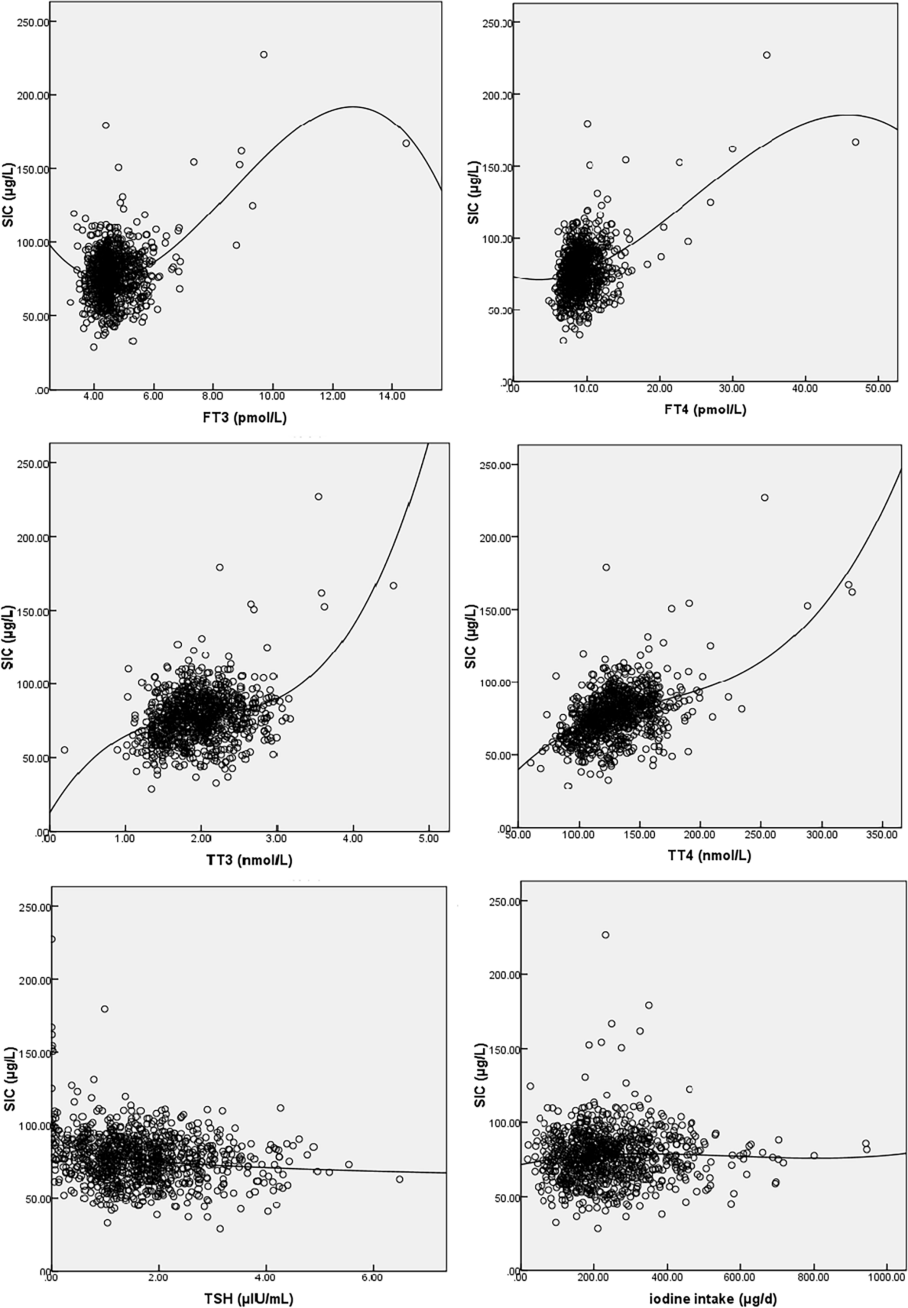
Fitted graphs for serum iodine (SI) and free triiodothyronine (FT_3_), free thyroxine (FT_4_), triiodothyronine (TT_3_), total thyroxine (TT_4_), thyroid-stimulating hormone (TSH), and iodine intake. The line represents regression trend. SI, serum iodine; FT_3_, free triiodothyronine; FT_4_, free thyroxine; TT_3_, triiodothyronine; TT_4_, total thyroxine; TSH, thyroid-stimulating hormone.

The correlation between maternal SIC and basic characteristics, UIC, thyroid function, and iodine nutrition variables is shown in **Supplementary Table 2**. As shown in **Figure 2**, SIC was nonlinearly correlated with TSH, FT_3_, FT_4_, TT_3_, TT_4_, and dietary iodine intake. The SIC was statistically significantly correlated with TSH (*r* = −0.141, *p* < 0.001), FT_3_ (*r* = 0.106, *p* = 0.001), FT_4_ (*r* = 0.236, *p* < 0.001), TT_3_ (*r* = 0.229, *p* < 0.001), TT_4_ (*r* = 0.433, *p* < 0.001), and dietary iodine intake (*r* = 0.068, *p* = 0.041). SIC showed strong positive correlations with TT_4_. In contrast, SIC was not significantly correlated with UIC.

### 3.4 Relationship between SI and iodine nutritional status

Characteristics of SIC in pregnant women with different iodine nutritional status in different trimesters are shown in **Table 3**. For all pregnant women surveyed, the median UIC was calculated on a district basis to determine the iodine nutrition level of pregnant women in that area. In 10 districts (counties), the median UIC of pregnant women was <150 μg/L, and they belonged to iodine-deficient areas; in 8 districts (counties), the median UI level of pregnant women was 150–249 μg/L, and they belonged to iodine-suitable areas. There was no statistical difference between groups (*Z* = −1.608, *p* = 0.108).

**Table 3:** Characteristics of SIC in pregnant women with different biological iodine nutritional status in different trimesters.

	Overall	T1	T2	T3
	*n* = 912	*n* = 215	*n* = 458	*n* = 239
Iodine deficiency				
SIC (μg/L)	77.84 (68.32, 87.25)	76.66 (66.51, 87.17)	78.75 (70.73, 87.93)	75.56 (66.21, 85.75)
*n* (%)	478 (52.4)	112 (12.3)	239 (26.2)	127 (13.9)
Iodine suitable				
SIC (μg/L)	76.38 (64.27, 87.43)	77.41 (65.82, 87.94)	76.41 (65.85, 88.44)	74.20 (61.64, 85.08)
*n* (%)	434 (47.6)	103 (11.3)	219 (24.0)	112 (12.3)
***p***	0.108	0.786	0.081	0.284
***Z***	−1.608	−0.272	−1.746	−1.071

T1, First trimester; T2, Second trimester; T3, Third trimester; SIC, Serum iodine concentration; Iodine deficiency, district median UIC <150 µg/L; Iodine suitable, district median UIC between 150 and 249 µg/L.

All values are reported as median (M) and interquartile range (IQR, P_25_–P_75_).

Significance between two iodine nutritional status using Mann–Whitney U test. Z is the test statistics. p-value significance set at p < 0.05.

### 3.5 Relationship between SI and iodine intake

Characteristics of maternal SIC at different levels of iodine intake in different trimesters are shown in **Table 4**. The highest proportion of pregnant women had appropriate iodine intake (72.59%), followed by iodine inadequate intake (25.66%). The results showed that there was a significant difference in SIC between inadequate intake (74.58 μg/L) and appropriate intake (77.92 μg/L) groups in pregnant women (*p* = 0.036).

**Table 4:** Characteristics of maternal SIC at different levels of iodine intake in different trimesters.

	Overall	T1	T2	T3
	*n* = 912	*n* = 215	*n* = 458	*n* = 239
Inadequate intake				
SIC (μg/L)	74.58 (64.10, 84.69) **^‡^**	72.92 (61.47, 83.80)	77.85 (68.22, 85.38)	71.44 (60.64, 78.45)
*n* (%)	234 (25.66)	60 (6.58)	113 (12.39)	61 (6.69)
Appropriate intake				
SIC (μg/L)	77.92 (67.60, 88.25)	77.56 (66.78, 88.6)	78.24 (68.68, 88.95)	77.52 (65.80, 85.51)
*n* (%)	662 (72.59)	151 (16.55)	335 (36.73)	176 (19.30)
Excessive intake				
SIC (μg/L)	78.25 (74.54, 82.31)	77.64 (71.99, 81.20)	78.49 (73.38, 81.22)	80.61 (77.99, 83.24)
*n* (%)	16 (1.75)	4 (0.44)	10 (1.10)	2 (0.22)
***p***	0.043*	0.258	0.641	0.061
***H***	6.315	2.711	0.890	5.584

T1, First trimester; T2, Second trimester; T3, Third trimester; SIC, Serum iodine concentration; Inadequate intake, intake < 160 µg/day; Appropriate intake, iodine intake between 160 and 600 µg/day; Excessive intake, iodine intake > 600 µg/L.

All values are reported as median (M) and interquartile range (IQR, P_25_–P_75_).

Significance between different iodine intakes in different trimesters using the Kruskal–Wallis H test. H is the test statistics. p-value significance set at p < 0.05.

**^‡^** Significant differences compared to Appropriate intake (H = −50.337, p = 0.036).

* p < 0.05.

The bold values mean that the p value is less than the set p value, and the difference is statistically significant.

### 3.6 Analysis of low or high SIC as a risk factor for thyroid diseases

The number of pregnant women with thyroid diseases at different gestation periods is shown in **Supplementary Table 3**, which shows the prevalence of thyroid disorders in pregnant women during different trimesters. Analyses of SIC <10% or >90% as a risk factor for thyroid diseases are shown in **Table 5**. When total SIC <10% or >90% was analyzed as risk factors for thyroid diseases, the 10th percentile of SI in pregnant women was 56.59 μg/L and the 90th percentile was 96.52 μg/L. Logistic regression results showed that low SIC was associated with hypothyroxinemia. OR for hypothyroxinemia is 3.18 (95% CI: 1.80–5.61) when SIC was less than the 10th percentile of the study sample. After adjusting for age, height, weight,and gestational factors, the OR remained statistically significant (adjusted OR = 3.14, *p* = 0.000 < 0.0001).

**Table 5:** Analysis of SIC<10% or >90% as a risk factor for thyroid diseases.

	Unadjusted			Adjusted*		
	OR	95% CI	*p*	OR	95% CI	*p*
SIC<P_10_						
Hypothyroidism	1.28	0.16, 10.49	0.820	1.31	0.16, 10.83	0.805
Subclinical hypothyroidism	1.38	0.31, 6.21	0.675	1,36	0.30, 6.18	0.689
Hypothyroxinemia	3.18	1.80, 5.61	<0.0001**	3.14	1.75, 5.66	<0.0001**
TGAb (+)	1.02	0.35, 2.94	0.971	1.04	0.36, 3.03	0.939
TPOAb (+)	1.22	0.54, 2.76	0.640	1.42	0.62, 3.28	0.409
Goiter	0	0	0.997	0	0	0.997
Thyroid nodules	0.90	0.35, 2.33	0.835	0.91	0.35, 2.35	0.841
SIC>P_90_						
Hypothyroidism	0	0	0.997	0	0	0.997
Subclinical hypothyroidism	0.63	0.08, 4.87	0.660	0.58	0.07, 4.47	0.598
Hypothyroxinemia	0.32	0.10, 1.04	0.058	0.33	0.10, 1.07	0.065
TGAb (+)	1.02	0.35, 2.94	0.971	0.72	0.21, 2.39	0.585
TPOAb (+)	1.01	0.42, 2.42	0.983	0.88	0.34, 2.28	0.787
Goiter	1.63	0.36, 7.49	0.527	2.11	0.44, 10.00	0.349
Thyroid nodules	0.70	0.25, 1.98	0.502	0.67	0.24, 1.92	0.459

SIC, Serum iodine concentration; CI, Confidence interval; TPOAb, Thyroid peroxidase antibody; TGAb, Thyroglobulin antibody.

*Adjusted for age, height, weight, and week of gestation factors.

**p < 0.0001.

The bold values mean that the p value is less than the set p value, and the difference is statistically significant.

### 3.7 Comparison of the diagnostic value of SI and UI

**Figure 3:**
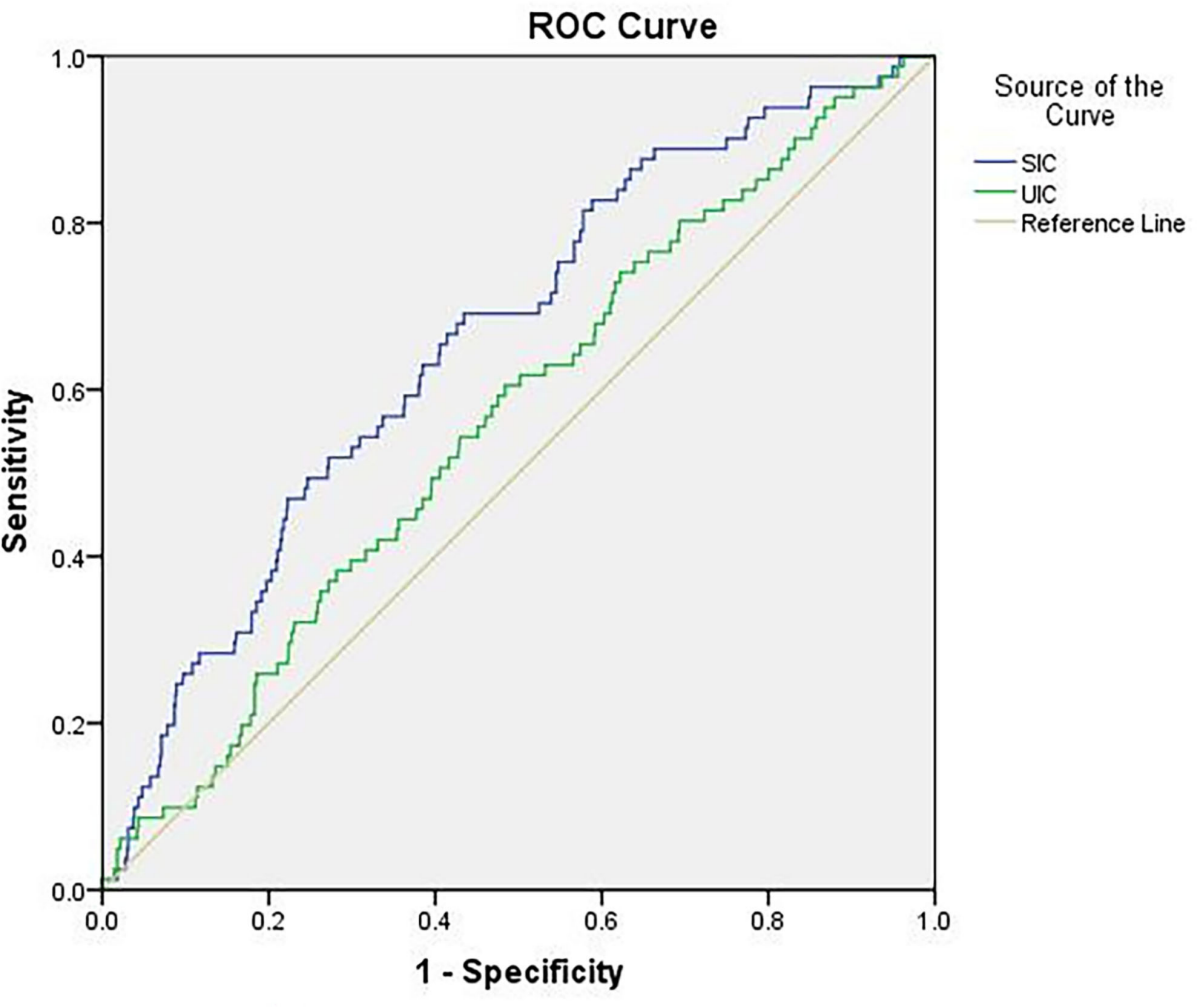
Receiver operating characteristic (ROC) curves for the diagnosis of hypothyroxinemia with serum and urinary iodine. CI, confidence interval; UIC, urinary iodine concentration; SIC, serum iodine concentration. The area under the ROC curve for SI was 0.66 (95% CI: 0.60–0.72, *p* < 0.0001) and UI was 0.57 (95% CI: 0.50–0.63, *p* = 0.053); SI had a higher diagnostic value than UI (*Z* = 2.110, *p* = 0.035).

ROC curves for the diagnosis of hypothyroxinemia with SI and UI are shown in **Figure 3**. The area under the ROC curve for the diagnosis of hypothyroxinemia using serum and urine iodine was 0.66 and 0.57, respectively. In the diagnosis of thyroid diseases, the diagnostic value of SI was significant (*p* < 0.0001). SI had a higher diagnostic value than urine iodine (*Z* = 2.110, *p* = 0.035).

### 3.8 Establishment of reference range of SIC

Characteristics of SIC in normal pregnant women by trimester are shown in **Table 6**. After excluding thyroid diseases, thyroid nodule, and goiter, 681 normal pregnant women were identified. The Kolmogorov–Smirnov test showed that the SI data were normally distributed (*Z* = 1.28, *p* = 0.074), so the 95% reference interval was determined using the normal distribution method. The reference range for SI in normal pregnant women is 45.03–112.44 µg/L. SI ranges from 36.93 to 122.52 µg/L in early pregnancy, 51.13–109.18 µg/L in mid-pregnancy, and 44.78–106.58 µg/L in late pregnancy. Comparisons between SI at different trimesters were analyzed by ANOVA. The mean SIC of pregnant women in the first (79.72 µg/L), second (80.15 µg/L), and third (75.68 µg/L) trimester was statistically different (*F* = 4.54, *p* = 0.011). The differences in SI between early and late pregnancy and between mid- and late pregnancy were statistically significant.

**Table 6:** Characteristics of SIC (μg/L) in normal pregnant women by trimester.

Groups	Number of cases	Mean	SD	95% reference interval
T1	169	79.72^*^	21.83	36.93–122.52
T2	312	80.15^†^	14.81	51.13–109.18
T3	200	75.68	15.76	44.78–106.58
Overall	681	78.73^‡^	17.20	45.03–112.44

T1, First trimester; T2, Second trimester; T3, Third trimester; SD, Standard deviation; SIC, serum iodine concentration.

Significance between groups using analysis of variance (ANOVA) test. p-value significance set at p < 0.05.

* Significant differences between T1 and T3 (p = 0.024).

† Significant differences between T2 and T3 (p = 0.004).

‡ Significant differences between trimesters (p = 0.011).

## 4 Discussion

Indicators and methods for assessing the iodine nutritional status of individuals are a controversial issue. The most commonly used tool is the UIC, which is influenced by a number of factors and has inconsistent results. Some studies have measured UIC by collecting 24-h urine samples to improve the accuracy of iodine nutrition assessment, but these methods are not applicable to large-scale epidemiological surveys (12). In addition, some studies have attempted to mitigate the effect of urine output on UIC by adjusting for urinary creatinine concentration (5, 32). However, factors such as age, ethnicity, skeletal muscle content, and protein intake can affect urinary creatinine concentration (33, 34). Therefore, the use of creatinine-adjusted UIC to assess individual iodine nutritional status remains highly controversial. In this context, the search for new individual iodine nutritional markers is crucial to accurately assess the iodine nutritional status of pregnant women and may provide a new scientific basis for personalized iodine supplementation in high-risk groups.

SI, as an important link of iodine metabolism in the body, is affected by iodine intake on the one hand, and on the other hand, thyroid dysfunction and thyroid disorders may be associated with SIC abnormalities. In this study, the applicability of SI as an indicator of individual iodine nutritional status was investigated from these two perspectives, which provides a basis for further evaluation of individual iodine nutritional status. In this study, pregnant women were found to be mildly deficient in iodine. There is a correlation between maternal SIC, thyroid function, and dietary iodine. When SICs were low, the percentage of hypothyroxinemia increased. Reference ranges during normal pregnancies were derived. SI appeared to be an indicator of a combination of iodine nutritional status and thyroid function.

In this study, the median UI of pregnant women was 148.40 μg/L < 150 μg/L, indicating mild iodine deficiency in pregnant women in Fujian Province, which was similar to our previous study and the results in coastal China (3, 17, 35, 36). Similar to provinces like Fujian, pregnant women in some developed countries also face iodine deficiency, such as those in Austria, Norway, and Sweden (37, 38). The causes of iodine deficiency in pregnant women are as follows: (1) a 50% increase in thyroid hormone secretion during pregnancy (10); (2) preferential administration of fetal iodine for fetal neurological development (39); (3) increased renal clearance of iodine during pregnancy (39); and (4) increased activity of the T_3_ deiodinase (39). In addition, vomiting during pregnancy may partially contribute to iodine loss. In addition, some pregnant women have opted for a low-salt diet to prevent edema and gestational hypertension (17). The UI of pregnant women in this study did not vary significantly, in contrast with the downward trend of SI. SIC was the lowest in the third trimester of pregnancy and lower than in the second trimester, which may be due to the continuous transport of large amounts of iodine ions from the pregnant woman to the fetus during pregnancy (31). The trend of changes in UIC in pregnant women is controversial: some studies suggest a decrease with increasing gestational weeks (40), some reports suggest an increase (41), and others report no change throughout pregnancy (42). The UIC of pregnant women in different trimesters did not vary significantly while SIC was significant, which match those observed in another study (36). Foreign studies have shown that iodine supplementation during pregnancy can prevent UIC from decreasing during pregnancy (43). Dietary iodine intake of pregnant women in this study was lower than the recommended intake (RNI, 230 μg/day), which is similar to the results of previous studies (19). This may be due to the fact that vomiting reaction affects the appetite of pregnant women. For pregnant women with mild iodine deficiency, iodine supplements are recommended. Iodine nutrition monitoring during pregnancy should be emphasized and iodine supplement is recommended according to local conditions.

This study showed that SI was positively correlated with TT_3_, TT_4_, and FT_4_, similar to the results of Songlin Yu et al. (16, 36), which can explain that SI is closely related to thyroid bioavailable iodine (12–15). SI is negatively correlated with TSH, the most sensitive indicator of abnormal thyroid function (44), indicating that SI may be a good biomarker for evaluating thyroid function. The increase in TSH is due to depletion of iodine stores in the thyroid, which may lead to hypothyroidism and elevated TSH levels (31, 45). Elevated TSH due to decreased SI has been reported to be associated with an increased risk of pre-term birth, placental abruption, fetal death, and neurodevelopmental impairment (46). SIC was associated with serum FT_4_ levels, but not associated with UIC. In this study, mean SIC in normal pregnant women increased in the second trimester of pregnancy, and then decreased in the third trimester of pregnancy. The difference between the SIC in normal pregnant women in the second trimester and the third trimester of pregnancy was statistically significant, which was similar to the results reported by Li et al. who reported that the SIC of pregnant women increased continuously from the 8th week of pregnancy to the 20th week of pregnancy and then decreased (5). This result also supports the speculation that the elevated SIC in pregnant women may be due to increased synthesis and secretion of thyroid hormones.

There is no significant correlation between SI and UI in our study, which is inconsistent with some research results (31, 47). The reason may be that the one-time UI in our study is not a relevant indicator of individual iodine nutritional status during pregnancy. This may be due to the fact that there are many factors affecting UIC in pregnant women such as water intake and sampling location, which may lead to individual UIC levels fluctuating greatly. For example, a Danish study found significant differences in UIC in samples from the same group of pregnant women collected at different locations (48). Another explanation is the modification of glomerular filtration and the ureter dilatation during pregnancy (5). In this study, there was a nonlinear relationship between SI and thyroid function, which was consistent with the study of Jin et al. (47). The study also found that SI was positively correlated with dietary iodine intake. Median SIC increased from the group with insufficient iodine intake to the group with excessive iodine intake, and the SIC among different dietary iodine intake groups was statistically significant. This suggests that SIC is useful in determining dietary intake. Inappropriate iodine intake may lead to abnormal SIC, which can affect thyroid function (36). The results above suggest that SIC is more useful and reliable in assessing the iodine nutritional status of individuals compared to UIC.

Previous studies have shown that thyroid disease can be caused by inadequate or excessive iodine intake (49), while few national and international epidemiological studies explored the effect of different SIC levels on thyroid diseases. Our study found that low SIC was associated with an increased percentage of hypothyroxinemia. Iodine deficiency can lead to maternal hypothyroxinemia (50, 51). Low SICs may be caused by insufficient iodine intake, which, in turn, leads to insufficient synthesis and secretion of thyroid hormones and ultimately lower serum thyroid hormone concentrations, which is in agreement with recent findings (31, 36). These results highlight the potential use of SIC as a biomarker of iodine nutritional status in individual pregnant women. Further studies are needed to confirm the relationship between SI and abnormal thyroid function, and more data are needed to support the interaction between SI and iodine nutritional status and thyroid hormone levels in women during pregnancy.

To further compare the diagnostic value of SIC and UIC for thyroid diseases, we analyzed the area under the curve (AUC) for SIC and UIC. The closer the area under the ROC curve is to 1, the higher the diagnostic value. We found that SIC has a good diagnostic value for thyroid diseases, and its accuracy is higher than that of UI. The results of this study are similar to other studies (47, 52). Therefore, SIC is a reflection of thyroid function, and maintaining SIC within the normal range is beneficial to thyroid health.

This study provides reference ranges for SICs in pregnant women in a mildly iodine-deficient region of southeastern coastal China. SIC levels during pregnancy have also been reported in some previous small studies (5, 13). In our study, the SIC of 78.73 μg/L in normal pregnant women was lower than the SIC in thyroid disorder patients in other iodine excess areas (47). The 95% reference intervals for SIC in the first, second, and third trimester, respectively, in this study were lower than in previous studies conducted in iodine-sufficient and iodine-deficient areas of China (31, 36). The 95% reference ranges for SIC provided by internationally recognized laboratories such as Mayo Clinic, Quest Diagnostics, and the WHO are 40–92 μg/L, 52–109 μg/L, and 45–90 μg/L, respectively (53). The range of SIC in normal pregnant women found in the present study was in agreement with previous works (47, 54) and was higher than the reference range of 36.0–79.3µg/L in non-pregnant women (16). This result can be explained by increased iodine absorption adaptive to an increased iodine demand during pregnancy, as well as higher consumption of iodine-rich foods in coastal areas. The elevated SIC in pregnant women may also be caused by a surge of thyroid hormones with TT_4_ levels being higher (55). Thus, our findings suggest that SIC may be a useful biomarker for assessing iodine nutritional status in individual pregnant women.

The main strengths of this study are as follows. Multistage sampling was used in this study and the sample was highly representative. The FFQ included the main food groups containing iodine, and the information collected by the questionnaire was adequate. This study, conducted on a large number of pregnant women, demonstrates that SI is an interesting biomarker of iodine status. Indeed, SI, as an important part of iodine metabolism in the body, is influenced by iodine intake on the one hand, and is associated with thyroid function and hypothyroxinemia on the other hand. This study also defines reference ranges of SICs in pregnant women. It is the first time that the above study has been conducted on pregnant women in Fujian Province.

Our study also has the following limitations: first, we only collected spot urine samples from pregnant women and lacked 24-h urine sample data and creatinine data. As our study is based on a large-scale epidemiological survey, these methods present greater challenges in terms of sample collection. Secondly, the study was a cross-sectional study with no repeated measurements in the same participants. In future studies, SIC and iodine intake should be tested throughout pregnancy using prospective study methods, and the reliability and applicability of SIC in assessing individual iodine status in the general population should be verified.

## Data availability statement

The data presented in this study are available on reasonable request from the corresponding author. The raw data cannot be shared publicly due to ethical restrictions because they contain potentially sensitive information. Requests to access the datasets should be directed to 18906913056@163.com.

## Ethics statement

The studies involving humans were approved by Medical Ethics Committee of Fujian Provincial Center for Disease Control and Prevention (No.2020032). The studies were conducted in accordance with the local legislation and institutional requirements. The participants provided their written informed consent to participate in this study.

## Author contributions

SJ: Formal Analysis, Investigation, Writing – original draft, Writing – review & editing. XW: Investigation, Writing – review & editing. JW: Investigation, Writing – review & editing. DC: Investigation, Writing – review & editing. ZC: Conceptualization, Investigation, Writing – review & editing.
