# Supplementary material for: Serum iodine concentration and its associations with thyroid function and dietary iodine in pregnant women in the southeast coast of China: a cross-sectional study
Source: Front Endocrinol (Lausanne). 2023 Nov 9;14:1289572. doi: 10.3389/fendo.2023.1289572 (PMC10665901; doi:10.3389/fendo.2023.1289572)
Supplement: Supplementary file 1 [file DataSheet_1.pdf]

Chemicals and instrumentation for iodine measurement

Determination of iodine in urine—As^{3+}-Ce^{4+}catalytic spectrophotometry [1]

Principle

Urine is digested with ammonium persulfate. Iodide is the catalyst in the reduction of ceric ammonium sulfate (yellow) to the cerous form (colourless), and is detected by the rate of color disappearance (Sand ell-Kolthoff reaction).

Equipment

1. Digestion temperature control heating device: constant temperature eliminator (temperature difference between wells≤1°C) (Isotemp® Dry Bath 145, Fisher Scientific).
2. Visible spectrophotometer, 1 cm colorimetric cup (SP-723, Shanghai Spectrum Instruments Co., Ltd).
3. Super thermostatic water bath (Polystat cc1, Huber).
4. Glass test tube: 15 mm×150 mm.
5. Stopwatch (Shanghai Xingzuan Stopwatch Co., Ltd).

Chemicals

Reagent specifications: Unless otherwise specified, all reagents used are analytically pure reagents, and the experimental water shall comply with GB/T 6682 Grade 2 water specification.

1. Ammonium persulfate ($(NH_4)_2S_2O_8$, M_r=228.2) (Sinopharm Shanghai Co., Ltd).
2. Sulfuric acid (H_2SO_4, $\rho_{20°C}$=1.84 g/mL), excellent purity (Sinopharm Shanghai Co., Ltd).
3. Arsenic trioxide (As_2O_3, Mr =197.8) (Sinopharm Shanghai Co., Ltd).
4. Sodium chloride, Superior Pure (Sinopharm Shanghai Co., Ltd).
5. Sodium hydroxide (NaOH, M_r =40.0) (Sinopharm Shanghai Co., Ltd).
6. Ammonium ceric sulfate ($Ce(NH_4)_4(SO_4)_4 \cdot 4H_2O$,$M_r$=623.6) (Sinopharm Shanghai Co., Ltd).
7. Potassium iodate (KIO_3, M_r =214.0), Reference reagent or standard material (National Institute of Metrology, China).

References

(1). National Health and Family Planning Commission of the People's Republic of China. Determination of iodine in urine—As^{3+}-Ce^{4+}catalytic spectrophotometry, WS/T 107.1–2016 (2016) (a Chinese health industry standard).

General test method in salt industry--Determination of iodine [2]

Principle

In acidic medium, iodate ions in the sample oxidize potassium iodide to precipitate elemental iodine, and titrate with sodium thiosulfate standard titration solution to determine iodine content.

Equipment

General Laboratory Instruments (such as Burette, Electronic Balance (SE202F, Ohaus)).

Chemicals

Reagent specifications: Unless otherwise stated, only reagents confirmed to be analytically pure and Grade 3 water as specified in GB/T 66822-2008 were used in the analysis.

1. Phosphoric acid solution (1 mol/L) (Sinopharm Shanghai Co., Ltd).
2. Potassium iodate solution (50g/L) (Sinopharm Shanghai Co., Ltd).
3. Potassium iodate standard solution (Sinopharm Shanghai Co., Ltd).
4. Standard titration solution of sodium thiosulfate (National Institute of Metrology, China).
5. Starch solution (5 g/L) (Sinopharm Shanghai Co., Ltd).

References

(2). Standardization Administration of China. General Test Method in Salt Industry -Determination of Iodic Ion, GB/T 13025.7–2012 (2012) (a Chinese national standards).

Determination of iodine in serum -Inductively coupled plasma mass spectrometry [3]

Principle

Serum samples were diluted with ascorbic acid-ammonium chloride-ethanolamine-ethanol mixture and introduced into inductively coupled plasma mass spectrometer. Atomized by a sampling system, argon gas is taken as carrier gas into an inductively coupled plasma torch. The iodine to be detected is converted into positive ions with positive charges through evaporation, dissociation, atomization and ionization, and enters the mass spectrometer through the ion collection system. The mass spectrometer separates the ions according to their mass-to-charge ratio, and the iodine ion count is detected by a detector. Iodine concentration in the sample is proportional to iodine ion count. The iodine concentration in serum is calculated from iodine standard curve by using rhenium as internal standard calibration.

Equipment

1. Inductively coupled plasma mass spectrometer (ICAP-RQ, Thermo Fisher Scientific).
2. Analytical balance: sensitivity 0.1 mg (EX225DZH/AD, Ohaus).
3. Vortex shaker (VORTEX 1, IKA).
4. Tube: polypropylene centrifuge tube (15 mL) or glass tube (15 mm x 120 mm);
5. Quantitative pipettes: 200 µL, 1 000 µL, 5 000 µL (Eppendorf).
6. Glass pipettes: 5 mL graduated pipettes and 10 mL single standard pipettes.
7. Volumetric flasks: 100 mL, 500 mL, 1 000 mL (Shanghai Shenbo Glass Instrument Co., Ltd).
8. Centrifuge.

Chemicals

1. Pure water, resistivity>18 MQ·cm, the rest of the indicators in line with the provisions of GB/T 6682 Grade 1 water.
2. Ascorbic acid ($C_6H_8O_6$), excellent purity (Sinopharm Shanghai Co., Ltd).
3. ammonium chloride (NH_4CI), excellent purity (Sinopharm Shanghai Co., Ltd).
4. Ethanolamine (C_2H_7NO), analytical purity (Sinopharm Shanghai Co., Ltd).
5. Anhydrous ethanol (C_2H_6O), excellent purity (Sinopharm Shanghai Co., Ltd).
6. Potassium iodate (KIO_3), reference reagent or standard substance (National Institute of Metrology, China).
7. Rhenium single element standard solution (Re, concentration 1000 µg/mL), standard substance (Guobiao (Beijing) Testing & Certification Co., Ltd).
8. Mass spectrometry tuning solution, recommended for Lithium (Li), cobalt (Co), yttrium(Y), indium (In), cerium (Ce), thallium (TI), uranium (U). Mixed tuning solution, the concentration of each element is 1 ug/L or 10 ug/L (Thermo scientific).
9. Argon gas (Ar), purity >99.999 percent (Fuzhou Liangtong Low Carbon

Technology Development Co., Ltd).

References

(3). People's Republic of China National Health Commission. Determination of Iodine in Serum-Inductively Coupled Plasma Mass Spectrometry, WS/T 783—2021 (2021) (a Chinese health industry standard).

Determination of iodine in drinking water by As^{3+}-Ce^{4+} catalytic spectrophotometry [4]

Principle

The catalytic effect of iodine on the arsenic-cerium redox reaction is utilized. The yellow Ce^{4+} is reduced to colourless Ce^{3+} in the reaction. The higher the iodine content, the faster the reaction rate and the less Ce^{4+} remaining. The iodine content was determined by controlling the reaction temperature and time and measuring the absorbance (A value) of the remaining Ce^{4+} in the system at a certain wavelength.

Equipment

1. Super thermostatic water bath (Polystat cc1, Huber).
2. Visible spectrophotometer, 1 cm colorimetric cup (SP-723, Shanghai Spectrum Instruments Co., Ltd).
3. Glass test tube: 15 mm×120 mm or 15 mm×150 mm.
4. Stopwatch (Shanghai Xingzuan Stopwatch Co., Ltd).

Chemicals

1. The purity of reagents used in this standard is analytically pure unless otherwise specified.
2. Concentrated sulfuric acid(H_2SO_4), excellent purity (Sinopharm Shanghai Co., Ltd).
3. Sodium hydroxide (NaOH), excellent purity (Sinopharm Shanghai Co., Ltd).
4. Arsenic trioxide (As_2O_3) (Sinopharm Shanghai Co., Ltd).
5. Sodium chloride (NaCl), excellent purity (Sinopharm Shanghai Co., Ltd).
6. Ammonium ceric sulfate ($Ce(NH_4)_4(SO_4)_4 \cdot 4H_2O$) (Sinopharm Shanghai Co., Ltd).
7. Potassium iodide (KI), excellent purity (Guobiao (Beijing) Testing & Certification Co., Ltd).
8. Ammonium persulfate ($(NH_4)_2S_2O_8$) (Sinopharm Shanghai Co., Ltd).
9. Deionized water (H_2O), the water shall comply with GB/T 6682 Grade 2 water Specification which conductivity ≤1.0 µs/cm.

References

(4). Wang H, Liu L, Li S, Gu Y, li X, Wang J, et al. Determination of Iodine in

Drinking Water by As^{3+}-Ce^{4+} Catalytic Spectrophotometry. Chin J Endemiol (2007) 26(3):4. (in Chinese).
