# Supplementary material for: Serum iodine concentration and its associations with thyroid function and dietary iodine in pregnant women in the southeast coast of China: a cross-sectional study
Source: Front Endocrinol (Lausanne). 2023 Nov 9;14:1289572. doi: 10.3389/fendo.2023.1289572 (PMC10665901; doi:10.3389/fendo.2023.1289572)
Supplement: Supplementary file 2 [file Table_1.docx]

Supplementary Material

# Supplementary Tables

**Supplementary Table 1. Diagnostic criteria for thyroid disorders in pregnant women.**

| **Thyroid disorders** | **TSH (mIU/L)** | **FT_4_ (pmol/L)** |
| --- | --- | --- |
| Subclinical hypothyroidism | Exceeds normal range | Within normal range |
| Hypothyroidism | Exceeds normal range | Less than normal range |
| Hypothyroxinemia | Within normal range | Less than normal range |

TSH, Thyroid stimulating hormone; FT_4_, Free thyroxine.

**Supplementary Table 2.** **Spearman's correlation between maternal SIC and basic characteristics, UIC, thyroid function, and iodine nutrition variables.**

| **Variables** | **Correlation coefficient (r)** | ***P*** | **95%*CI*** |
| --- | --- | --- | --- |
| **Age (years)** | -0.036 | 0.280 | -0.102, 0.031 |
| **Height(m)** | 0.029 | 0.385 | -0.038, 0.095 |
| **Body weight (kg)** | -0.041 | 0.228 | -0.108, 0.027 |
| **BMI (kg/m^2^)** | -0.057 | 0.088 | -0.125, 0.011 |
| **Gestational weeks (weeks)** | -0.053 | 0.110 | -0.119, 0.014 |
| **UIC (μg/L)** | 0.003 | 0.935 | -0.064, 0.070 |
| **FT_3_ (pmol/L)** | 0.106* | 0.001 | 0.040, 0.172 |
| **FT_4_ (pmol/L)** | 0.236** | <0.001 | 0.172, 0.299 |
| **TT_3_ (nmol/L)** | 0.229** | <0.001 | 0.165, 0.292 |
| **TT_4_ (nmol/L)** | 0.433** | <0.001 | 0.377, 0.486 |
| **TSH (μIU/mL)** | -0.141** | <0.001 | -0.205, -0.074 |
| **Tg (ng/mL)** | 0.002 | 0.960 | -0.065, 0.068 |
| **Iodine intake (µg/d)** | 0.068* | 0.041 | 0.001, 0.134 |

CI, Confidence interval; SIC, Serum iodine concentration; BMI, Body mass index; UIC, Urinary iodine concentration; FT_3_, Free triiodothyronine; FT_4_, Free thyroxine; TT_3_, Triiodothyronine; TT_4_, Total thyroxine; TSH, thyroid stimulating hormone; Tg, Thyroglobulin.

* *P*<0.05, ** *P*<0.001.

**Supplementary Table 3.** **Number of pregnant women with thyroid diseases at different gestation periods n (%).**

| **Variables** | **T1** | **T2** | **T3** | **Total** |
| --- | --- | --- | --- | --- |
| **Subclinical hypothyroidism** | 5(0.55) | 8(0.88) | 2(0.22) | 15(1.65) |
| **Hypothyroidism** | 4(0.44) | 3(0.33) | 1(0.11) | 8(0.88) |
| **Hypothyroxinemia** | 0(0) | 68(7.46) | 13(1.43) | 81(8.89) |
| **TPOAb (+)** | 23(2.52) | 30(3.29) | 6(0.66) | 59(6.47) |
| **TGAb (+)** | 11(1.21) | 21(2.30) | 7(0.77) | 39(4.28) |
| **Thyroid nodules** | 16(1.75) | 28(3.07) | 10(1.10) | 54(5.92) |
| **Goiter** | 1(0.11) | 9(0.99) | 3(0.33) | 13(1.43) |

T1, First trimester; T2, Second trimester; T3, third trimester; TPOAb, Thyroid peroxidase antibody; TGAb, Thyroglobulin antibody.
